# Surgical management of complicated gynecomastia (associated with foreign body injection) with single-port 3-dimensional videoscope-assisted endoscopic subcutaneous mastectomy and concurrent liposuction: A case report

**DOI:** 10.1097/MD.0000000000025962

**Published:** 2021-06-04

**Authors:** Tzu-Cheng Wen, Hung-Wen Lai, Chi Wei Mok, Shou-Tung Chen, Dar-Ren Chen, Shou-Jen Kuo

**Affiliations:** a Endoscopy & Oncoplastic Breast Surgery Center, Changhua Christian Hospital, Changhua; b Division of General Surgery, Changhua Christian Hospital, Changhua; c Comprehensive Breast Cancer Center, Changhua Christian Hospital, Changhua; d Minimal invasive surgery research center, Changhua Christian Hospital, Changhua; e Kaohsiung Medical University, Kaohsiung; f Division of Breast Surgery, Yuanlin Christian Hospital, Yuanlin; g School of Medicine, Chung Shan Medical University, Taichung; h School of Medicine, National Yang Ming University, Taipei, Taiwan; i Division of Breast Surgery, Department of Surgery, Changi General Hospital; j Singhealth Duke-NUS Breast Centre, Singapore Health Services, Singapore.

**Keywords:** 3 dimensional, endoscopic mastectomy, gynecomastia, liposuction, subcutaneous mastectomy

## Abstract

**Rationale:**

Gynecomastia is a common benign breast disorder in men. Surgical management of gynecomastia includes that of a subcutaneous mastectomy with or without concurrent liposuction. Herein, the authors presented a case of complicated gynecomastia (gynecomastia with concurrent foreign body injection) which was successfully managed with an innovative technique that offered acceptable operative time, minimal complications, good recovery and satisfactory aesthetic outcome.

**Patient Concerns:**

A 39-year-old Taiwanese man who developed gynecomastia along with self-injection of foreign body (salad oil) over the past 10 years for breast enlargement presented as symptomatic bilateral breast lumps.

**Diagnosis:**

Bedside sonography revealed multiple large droplets of oil in the subcutaneous tissue bilaterally, resembling cystic lesions.

**Intervention:**

Bilateral single-port 3-dimensional videoscope-assisted endoscopic subcutaneous mastectomy was performed after bilateral breast liposuction. Operative findings include bilateral gynecomastia and previous bilateral breast foreign body material. The total weight of lipoaspirate was 400 grams and 300 grams for right and left side respectively. Subcutaneous mastectomy specimen weight was 820 grams and 661 grams for right and left breast tissue.

**Outcomes:**

Operative duration was 315 minutes and intraoperative blood loss at 150 ml. Patient was discharged 2 days after the operation, and subsequent follow up ultrasound showed complete removal of foreign bodies and fibrotic breast tissue. Patient was satisfied with the post-operative aesthetic outcomes.

**Lessons:**

Single-port 3-dimensional videoscope-assisted endoscopic subcutaneous mastectomy with concurrent liposuction is a promising and safe surgical option for patient with complicated gynecomastia and severe fibrosis.

## 1 Introduction

Gynecomastia is a benign condition seen in pubertal men and by far the most common male breast disorder with incidence ranging from 40% to 55%.^[10]^ In suitable cases, operative management has been shown to be preferred treatment with high satisfaction.^[11]^ Ohyama et al reported the first successful endoscopic assisted trans-axillary mastectomy for the removal of glandular tissue in gynecomastia.^[9]^ Ever since then, there has been numerous case studies reporting on the safety and feasibility of endoscopic resection of the excessive breast tissue^[3,12,14]^ with comparative outcomes to traditional surgery or liposuction.^[5]^

Herein, we reported a case of complicated gynaecomastia from chronic foreign body (salad oil) injection, resulting in severe breast tissue fibrosis accompanied with chronic inflammation. This patient underwent a single sitting liposuction followed by single-port 3 dimensional (3D) videoscope assisted endoscopic subcutaneous mastectomy (E-SM) for excision of gynecomastia and removal of foreign body. This procedure demonstrated acceptable operative time with minimal morbidity, good recovery, and satisfactory aesthetic outcomes.

### 1.1 Case report

**Figure 1:**
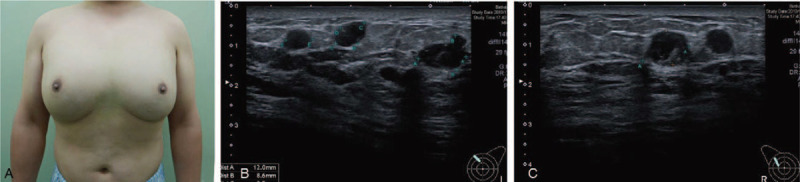
Preoperative picture and sonography examinations. A. Preoperative anterior view. B. Preoperative left breast sonography. C. Preoperative right breast sonography.

The authors presented a case of a 39-year-old gentleman who was otherwise healthy with no significant co-morbidities. He was seen at Changhua Christian Hospital with a complaint of bilateral breast lumps. Upon physical examination, large, uneven breasts were noted (Fig. 1A), and sonography revealed multiple large droplets of oil inside subcutaneous tissue bilaterally, resembling cystic lesions (Fig. 1B, C). In view of the above findings and the lack of any previous surgical intervention, a thorough history from the patient subsequently revealed a history of self-injection of salad oil, about 100 ml each time in both breasts, about 10 times over the past 10 years in his attempt to enlarge his breasts, mainly due to psychological reasons. As he became increasing dissatisfied with the appearance of both breasts, he decided to seek medical advice for surgical removal. Other than foreign body injection, patient also had Simon Grade 2 gynecomastia.^[11]^

## 2 Operative methods

Endoscopic operative resection of the excessive breast tissue was performed in January 2020. Preoperative marking was performed with the patient in the standing position (Figs. 1A, 4A-C). After induction, patient was placed in a supine position with ipsilateral arm abducted at 90° (Fig. 3A). The ipsilateral shoulder was then elevated to 30° to facilitate access. A tumescent solution containing lactated Ringer's solution with lidocaine 0.05% and epinephrine 1:1,000,000 was injected subcutaneously into the whole breast to minimize bleeding. An approximately 4–5 cm oblique axillary incision was made over the extramammary region near the anterior axillary line. Ultrasonography-assisted bilateral liposuctions were performed prior to endoscopic subcutaneous mastectomies in order to create subcutaneous tunnel between the skin flap and breast glandular tissue as well as to relieve fibrotic adhesions due to previous multiple episodes of foreign body injection. The total weight of lipoaspirate was 400 grams and 300 grams for right and left side respectively (Fig. 3B-D).

**Figure 2:**
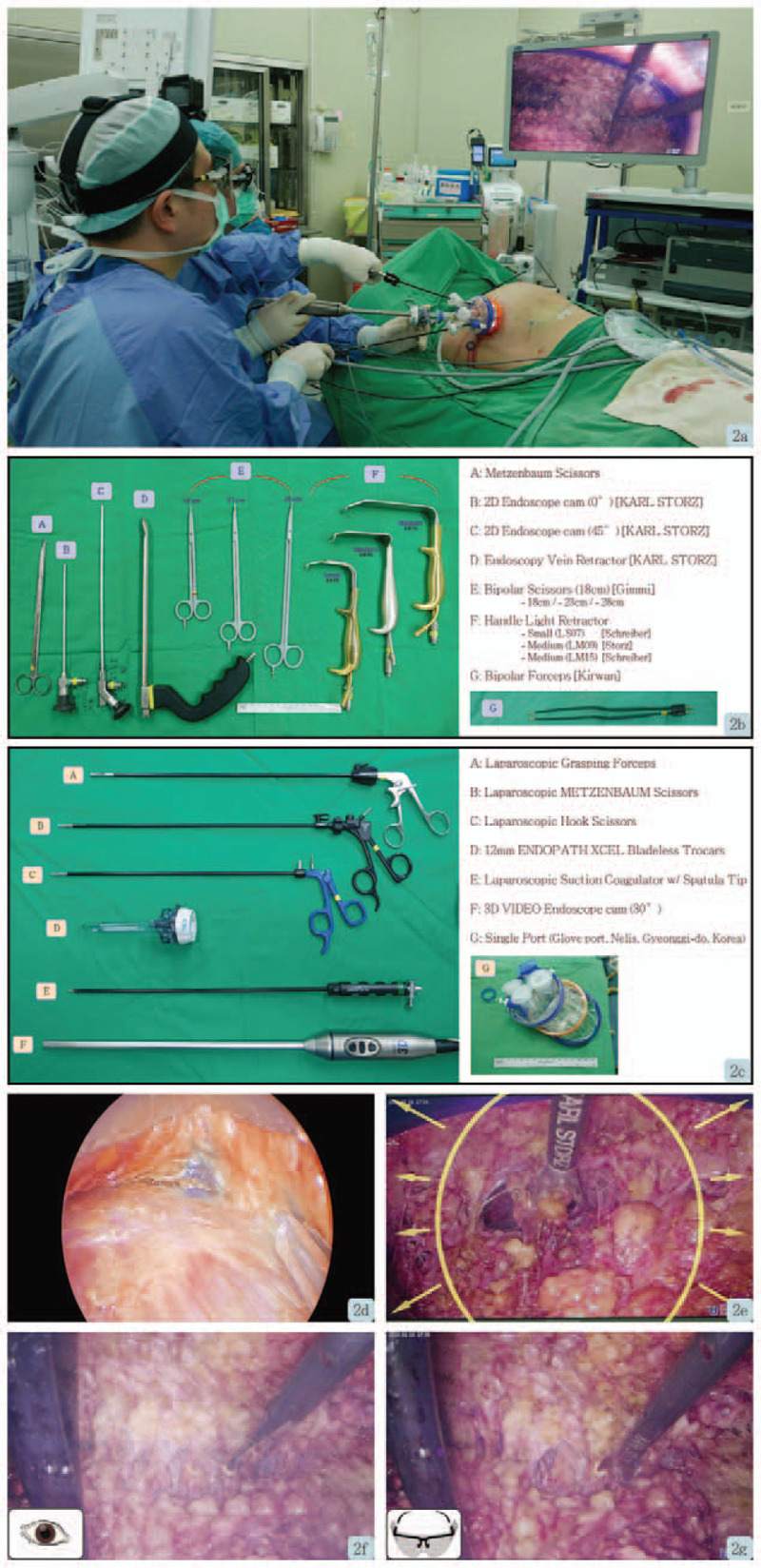
Photo demonstrating operations and instruments used for single port 3 dimensional videoscope assisted endoscopic subcutaneous mastectomy. A. Position of operator and assistant while operation. B. Operative instruments for bilateral breast liposuction prior to endoscopic mastectomy. C. Operative instruments for single-port 3D endoscopic-assisted subcutaneous mastectomy. D, 2E. Operative view of 2-dimensional (2D) endoscopy. F. Operative view of 3D endoscopy, under bare eyes. G. Operative view of 3D endoscopy, under 3D glasses.

The detailed technique of single port 3D videoscope assisted endoscopic nipple sparing mastectomy was published in a previous study^[6]^ hence a summary of the technique was provided here. To create the working space for the placement of the single port (Glove Port; Nelis, Gyeonggi-do, Korea), the subcutaneous flap was dissected under direct vision for 3–4 cm. After port placement, carbon dioxide insufflation with air pressure at 8 mm Hg was performed to create space for mastectomy. A 30° 10-mm diameter camera TIPCAM 1 S 3D VIDEO Endoscope (KARL STORZ, Germany) was used. The position of the endoscopic instruments, surgeon, anesthetist, and patient are shown in (Fig. 2A) Dissection was carried out with laparoscopic curved Metzenbaum scissors (KARL STORZ, Germany), whereas counter-traction was carried out with a laparoscopic grasping forceps (Fig. 2B-C).

**Figure 3:**
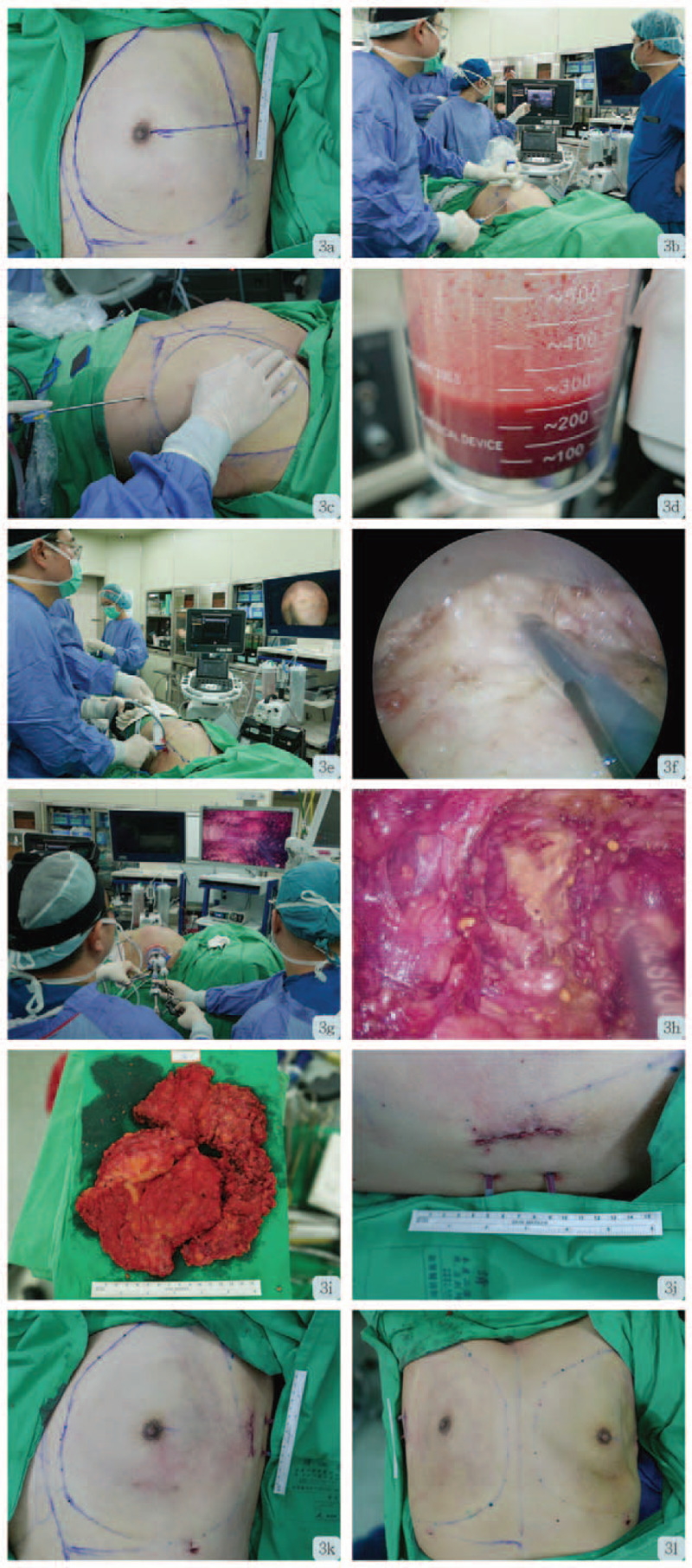
Operative techniques and procedural steps (using left mastectomy as example). A. Preoperative anterior view with markings of breast tissue verge. B, C. Position of operative team while performing left breast liposuction assisted with intra-operative sonography. D. Suctioned tissue of left breast. E, F. Position of operative team while performing 2D endoscopic resection and operative view of 2D endoscopy. G, H. Position of operative team while performing 3D endoscopic resection and operative view of 3D endoscopy. I. Resected breast tissue. J. Incision wound with placed drainage tube. K, L. Postoperative anterior view immediately after mastectomy.

The port placement of 3D scope, scissors, or grasping forceps was flexible and could be adjusted as necessary during the operation. To facilitate skin flap dissection, tunneling technique was applied, and the septum between the skin flap and parenchyma was subsequently dissected using laparoscopic Metzenbaum scissors. During skin flap dissection, a 30° upward facing 3D endoscope with reverse 180° imaging was used to produce a clear 3D vision (Fig. 2D-G). The angle and field of vision could be adjusted with either upward, downward, or reverse motion of the image by the 3D endoscope when necessary. For dissection near or beneath the nipple areolar complex (NAC) region, laparoscopic hook scissor (Snowden Pencer; BD) was used to cut the dense glandular tissue. (Fig. 3E-H)

After completion of superficial skin flap dissection, the peripheral and posterior dissection was carried out. Perforator vessels were clearly identified and adequately coagulated to achieve hemostasis. After the completion of bilateral dissection, the breast specimen was removed in pieces (to prevent enlarged surgical wound) and removed through the axillary incision. (Fig. 3I) 820 grams and 661 grams of the right and left breast tissue were resected respectively. Two 10 Fr Jackson-Pratt drains were placed over right and left breasts, separately. (Fig. 3J) The wounds were closed in layers with 3-0 polysorb and 4-0 monocryl. (Fig. 3K, L)

## 3 Outcomes

**Figure 4:**
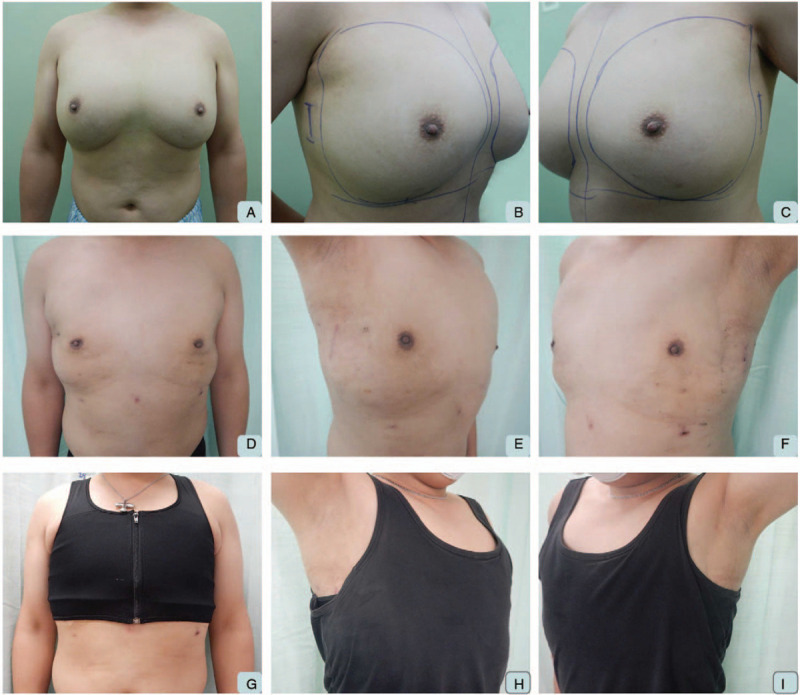
Postoperative photos. A. Preoperative anterior view. B. Preoperative right oblique view. C. Preoperative left oblique view. D. Postoperative anterior view without clothing. E. Postoperative right oblique view without clothing. F. Postoperative left oblique view without clothing. G. Postoperative anterior view with pressure garment. H. Postoperative right oblique view with vest and well-hidden operative scar. I. Postoperative left oblique view with vest and well-hidden operative scar.

Total operative time was 315 minutes and blood loss was around 150 ml. Patient was discharged 2 days after the operation, and post-operative recovery was uneventful. Serial sonography showed complete removal of foreign bodies and fibrotic breast tissue without seroma accumulation. At his 3-month post-operative outpatient follow-up, wounds had completely healed without retraction or redundant skin (Fig. 4). Patient was satisfied with the whole treatment process and his current appearance.

## 4 Discussion

The authors reported this unusual case with excessive foreign body (salad oil) injection into the breast tissue, resulting in a tissue expansion effect comparable to simple gynecomastia (Figs. 1, 3 and 4). After first graded by Simon in 1973,^[11]^ there were several classifications of gynecomastia according to different definitions,^[12]^ and this patient presented as mixed/complicated gynecomastia with both hypertrophy of fat and glandular tissue as well as foreign body (salad oil) injection, thereby posing great challenge in the management. A total of 1220 gm of right breast tissue and 961 gm of left breast tissue was resected using single-port 3D E-SM and liposuction. The post-operative recovery was uneventful and patient was satisfied with the aesthetic outcomes of the small and hidden scars as well as the symmetrical breast contour (Fig. 4).

Surgical intervention remained the most definite treatment in correcting breast contour,^[10]^ especially in Simon Grade 1-2 gynecomastia. High grade gynecomastia is best treated with conventional operation or liposuction but this can either result in a large operative scar or asymmetries from residual breast tissue.^[3]^ There had been a few case reports of endoscopic subcutaneous mastectomy in higher-grade gynecomastia demonstrating better outcomes with less scarring, short operative time, minimal complications, and good esthetic results.^[3,12]^ Furthermore, a single port incision results in higher aesthetic outcomes even though a longer operative time might be needed.^[7]^ Patients who underwent gynecomastia subcutaneous mastectomy through endoscopic axillary approach had higher scar satisfaction with better postoperative outcomes.^[13,14]^

Repetitive injection of foreign body for the augmentation of body contour had been reported since the late 19th century,^[1]^ and still occasionally noted in recent days. Non-medical foreign body injection might induce tissue reactions related to the local inflammation and subsequent scarring, while systemic complications may lead to granulomatous hepatitis, pneumonitis, systemic embolism, autoimmune disease, and sometimes even lethal.^[4,8]^ The resultant scarring and fibrosis can prove to be a challenge in the surgical dissection as it most often than not leads to difficult identification of clear and distinct dissection planes (Figs. 1–3).

To solve this problem as well as to decrease the risk of NAC ischemia/necrosis risk, we performed tumescent injection followed by liposuction to release the adhesion bands and fibrosis related to previous repeat foreign body injection (Fig. 3). Thereafter, we use single port with air inflation system for expansion of surgical field (Figs. 2 and 3). The adoption of 3D videoscope increased the visual acuity and depth perception,^[6]^ which translated to higher surgical performance. The wound location was at the lateral chest at anterior axillary line at the level of NAC (Fig. 3A and 4B). Sparing of an areolar incision,^[2]^ which was usually used in conventional surgical approach, could reduce the risk of NAC ischemia/necrosis injury, ensure that the scar is hidden, and improve aesthetic results. Comparing to trans-axillary approach, this incision results in a superior aesthetic outcomes (Figure 4D-I), and higher patient's satisfaction.

There are, however, some limitations of this technique. First, the single access approach with videoscope and rigid endoscopic instruments might possess technical challenges for beginner in their initial learning curve. Furthermore, the longer operation time required and getting used to the need for more instruments in the initial learning curve were another two factors to overcome. From our current case experience or other reported study,^[5,15]^ single-port E-SM for gynecomastia was safe and feasible, appropriate as a routine surgical treatment alternative for gynecomastia, and adopting 3D videoscope provided a significant improvement in terms of visual acuity and depth perception.

## 5 Conclusion

In conclusion, single-port 3D E-SM was a good alternative surgical treatment for gynecomastia, which was safe, feasible, and associated with small and inconspicuous hidden scars. The combination of liposuction could be applied in difficult or complicated gynecomastia as demonstrated in this report.

## Acknowledgment

The authors would like to thank Chin-Mei Tai, Yun-Ting Chang, and Ying-Ru Lai for the assistance in this study.

## Author contributions

**Supervision:** Hung-Wen Lai, Chi Wei Mok, Shou-Tung Chen, Dar-Ren Chen, Shou-Jen Kuo.

**Writing – original draft:** Tzu-Cheng Wen.

**Writing – review & editing:** Hung-Wen Lai, Chi Wei Mok.
